# Computational Prodrug Design Methodology for Liposome Formulability Enhancement of Small-Molecule APIs

**DOI:** 10.1021/acs.molpharmaceut.2c01078

**Published:** 2023-03-20

**Authors:** Martin Balouch, Kateřina Storchmannová, František Štěpánek, Karel Berka

**Affiliations:** † Department of Chemical Engineering, University of Chemistry and Technology, Prague, Technická 3, 166 28 Prague 6, Czech Republic; ‡ Department of Physical Chemistry, Faculty of Science, Palacký University Olomouc, 17. listopadu 12, 771 46 Olomouc, Czech Republic

**Keywords:** lipid bilayer, permeability, partitioning coefficient, prodrug, COSMOperm

## Abstract

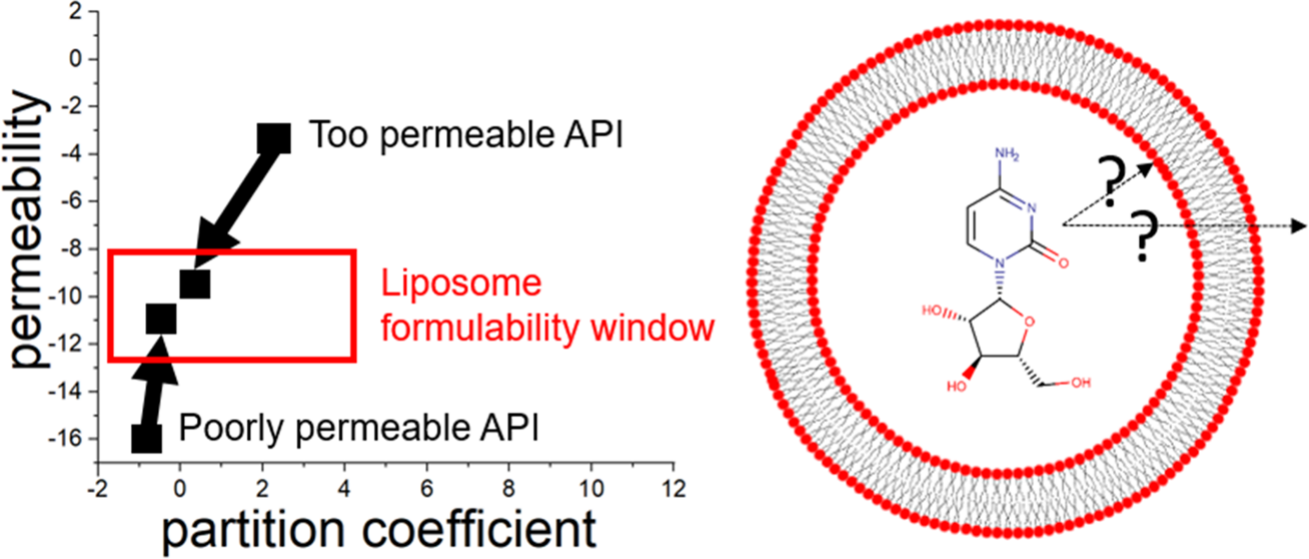

Encapsulation into liposomes is a formulation strategy that can improve efficacy and reduce side effects of active pharmaceutical ingredients (APIs) that exhibit poor biodistribution or pharmacokinetics when administered alone. However, many APIs are unsuitable for liposomal formulations intended for parenteral administration due to their inherent physicochemical properties—lipid bilayer permeability and water–lipid equilibrium partitioning coefficient. Too high permeability results in premature leakage from liposomes, while too low permeability means the API is not able to pass across biological barriers. There are several options for solving this issue: (i) change of the lipid bilayer composition, (ii) addition of a permeability enhancer, or (iii) modification of the chemical structure of the API to design a prodrug. The latter approach was taken in the present work, and the effect of small changes in the molecular structure of the API on its permeation rate across a lipidic bilayer was systematically explored utilizing computer simulations. An in silico methodology for prodrug design based on the COSMOperm approach has been proposed and applied to four APIs (abiraterone, cytarabine, 5-fluorouracil, and paliperidone). It is shown that the addition of aliphatic hydrocarbon chains via ester or amide bonds can render the molecule more lipophilic and increase its permeability by approximately 1 order of magnitude for each 2 carbon atoms added, while the formation of fructose adducts can provide a more hydrophilic character to the molecule and reduce its lipid partitioning. While partitioning was found to depend only on the size and type of the added group, permeability was found to depend also on the added group location. Overall, it has been shown that both permeability and lipid partitioning coefficient can be systematically shifted into the desired liposome formulability window by appropriate group contributions to the parental drug. This can significantly increase the portfolio of APIs for which liposome or lipid nanoparticle formulations become feasible.

## 1 Introduction

Since the first liposomal formulation of an active pharmaceutical ingredient (API) was approved in 1995,^1^ lipid nanoformulations have rapidly developed, leading to the recent rollout of mRNA vaccines.^2,3^ The encapsulation of macromolecules such as antibodies or nucleic acids^4^ within liposomal structures is facilitated mainly by combining steric factors and electrostatic interactions with charged lipids. Regarding small molecules, formulability into liposomes for parenteral application strongly depends on the lipid/water partitioning and the permeation rate across the liposome bilayer, which is unfavorable for many APIs. Only about 20 liposomal formulations of small-molecule APIs are currently approved in the EU and the U.S.A. Liposomal formulations in clinical trials are often only previously used APIs in different combinations or strengths^5^ rather than new chemical entities. The development of liposomal formulations of new APIs is limited because many molecules in their native form are inherently unsuitable for liposome encapsulation and release.

**Figure 1:**
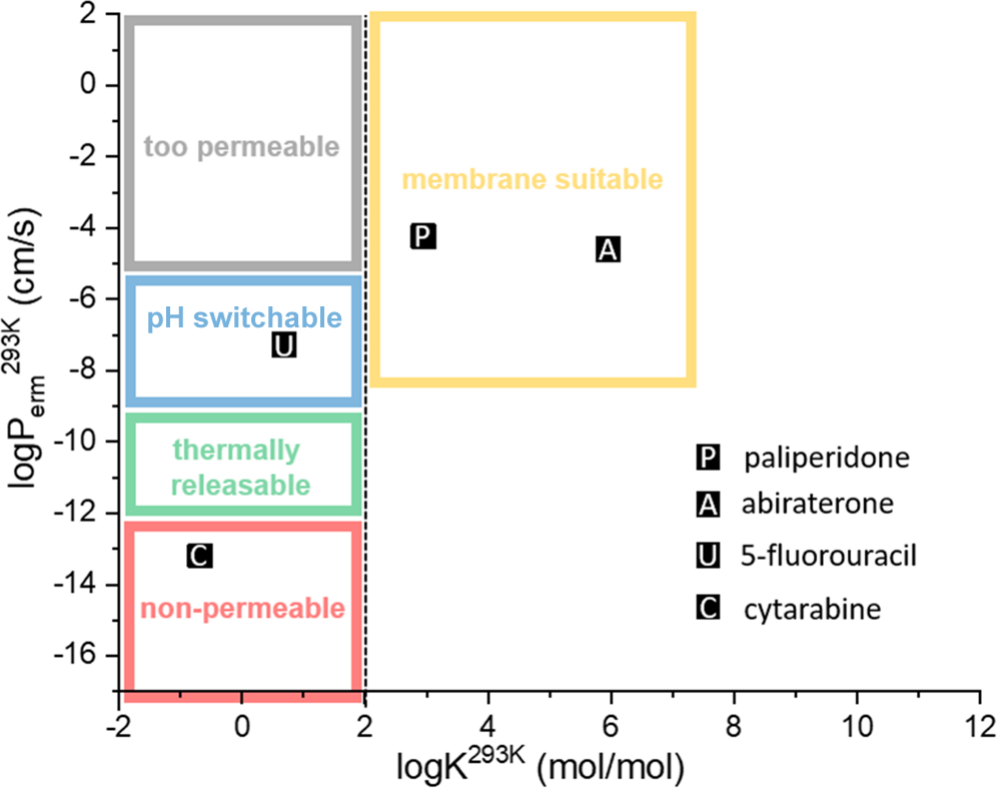
Parametric space showing the sorting mechanism for LBCS classification, where log *K* is the membrane/water partition coefficient and log *P*_erm_ is the permeation rate through the liposome membrane at the designated temperature. Gray bin: APIs too permeable for liposome formulation; blue bin: neutral APIs too permeable but possible to move to the green bin if ionizable in liposome cavity by pH; green bin: APIs suitable for liposome formulation and even for thermally induced release; red bin: APIs suitable for liposome formulation but not for thermally induced release; yellow bin: lipophilic APIs suitable for entrapment in the liposome membrane. Positions of the four APIs investigated in this work are marked by the squares.

An API′s partitioning and permeation properties need to be properly balanced to be suitable for liposome formulation intended for parenteral administration. The permeability across the liposome membrane should be sufficiently high to allow the API to be released from the liposomes in the target tissue or cell but sufficiently low to avoid premature spontaneous leakage. The lipid/water partitioning coefficient should be sufficiently high to allow the API to overcome the energy barrier represented by the lipid bilayer but sufficiently low to prevent the API from being trapped in the membrane. The phenomena of API partitioning and permeability were investigated computationally and validated experimentally in our recent publication.^6^ Systematic rules for the formulability of small-molecule APIs in liposomes have been proposed using the liposome biochemical classification system (LBCS). LBCS was designed as a two-dimensional (2D) diagram with approximative areas for the partitioning and permeation constants for each pair of API and lipid bilayer composition that enable or prevent successful liposome formulation. The meaning of these areas is explained in Figure 1.

To change the API/membrane behavior (i.e., the API position in the LBCS parametric space), there are, in principle, three options: (i) changing the membrane composition, (ii) using a permeation enhancer, or (iii) modifying the structure of the API. The membrane composition can be changed, e.g., by adding cholesterol or specific phospholipids to influence the membrane phase transition temperature and lipid bilayer structure. The key characteristics governing permeability through membranes were published recently.^7^ The bilayer thickness, the lipid phase, and the sterol type were found to play the main role. In the specific case of ceramide bilayers, the length of the ceramide was found to play a crucial role as well.^8^ Quantitatively, a change of lipid bilayer composition is generally suitable for fine-tuning the permeation/partitioning values but not for achieving profound, orders-of-magnitude shifts within the LBCS diagram for a given API.

Permeation enhancers can achieve more dramatic shifts in permeability, substances added to the API–lipid bilayer system to modify the permeation rate^9,10^ by various mechanisms such as the disruption of phospholipid packing in the bilayer, solubilization of the API,^11,12^ or facilitating transport.^13^ Permeation enhancers are mostly studied in the context of skin permeation, where the lipidic membrane is easily accessible. Also, there are studies into permeation enhancers for the oral route of administration, e.g., for improving the oral administration of peptides.^14^ Examples of permeability enhancers for oral peptide administration include citric acid, sodium caprate, dodecyl-β-d-maltopyranoside, and others.^15^

The third option for affecting permeation and partitioning properties is to modify the molecular structure of the API itself, i.e., to design a prodrug. In the pharmaceutical practice, prodrugs are used for various reasons, such as to modify an API′s solubility or dissolution rate. Examples include the antipsychotic drug paliperidone, whose ester paliperidone palmitate^16^ controls the dissolution rate in an intramuscular depot formulation. Another example is abiraterone, an anticancer drug whose ester abiraterone acetate^17^ is used for improving solubility in the gastrointestinal tract. However, there are no reports of designing prodrugs specifically to enable liposomal formulations of small-molecule drugs. When considering the prodrug design, there comes a question of whether and how the API can be modified to obtain the desired lipid/water partitioning and liposome bilayer permeation behavior. Generally, the partition coefficient and permeation rate are considered to be strongly correlated based on the Meyer–Overton rule.^18^ They correlate strongly in the case of very simple molecules^19^ (molar mass around and under 50 Da), but in the case of more complex molecules, this approximation works poorly.^20^ Moreover, the combination of prodrug synthesis and permeation enhancement is also possible. The key principle is the modification of API and its incorporation into the liposome.^21^ This approach includes diglyceride conjugate of doxorubicin^22^ or cholesterol conjugate of topoisomerase I inhibitor 7-ethyl-10-hydroxycamptothecin.^23^

Therefore, the present work aims to propose a general methodology for computational prodrug design by systematically investigating the relationship between API molecular structure modification and its position in the LBSC diagram. To this end, four APIs with different initial positions in the LBSC diagram have been chosen (cytarabine, fluorouracil, paliperidone, abiraterone) and systematically modified by adding either lipophilic or hydrophilic groups of different properties (e.g., length of acyl chain) or position. These molecules represent general situations where an API is initially unsuitable for liposomal formulation either because of too low or too high permeability or due to an unsuitable partitioning coefficient, as shown in Figure 1. Apart from their position in the LBCS diagram, the rationale for choosing these four APIs is that they all provide opportunities for substituting functional groups in their molecular structure without impacting the pharmacophore responsible for their biological action. In the case of paliperidone^16^ and abiraterone,^17^ the ability to form ester prodrugs without negatively influencing bioactivity has even been shown experimentally, and these ester prodrugs are successfully marketed and clinically used. We aim to provide more general guidelines for rational prodrug design by systematically exploring the relationship between API molecular structure modification and its position in the permeability–partitioning diagram.

## 2 Materials and Methods

### 2.1 Preparation of Molecular Structures

**Figure 2:**
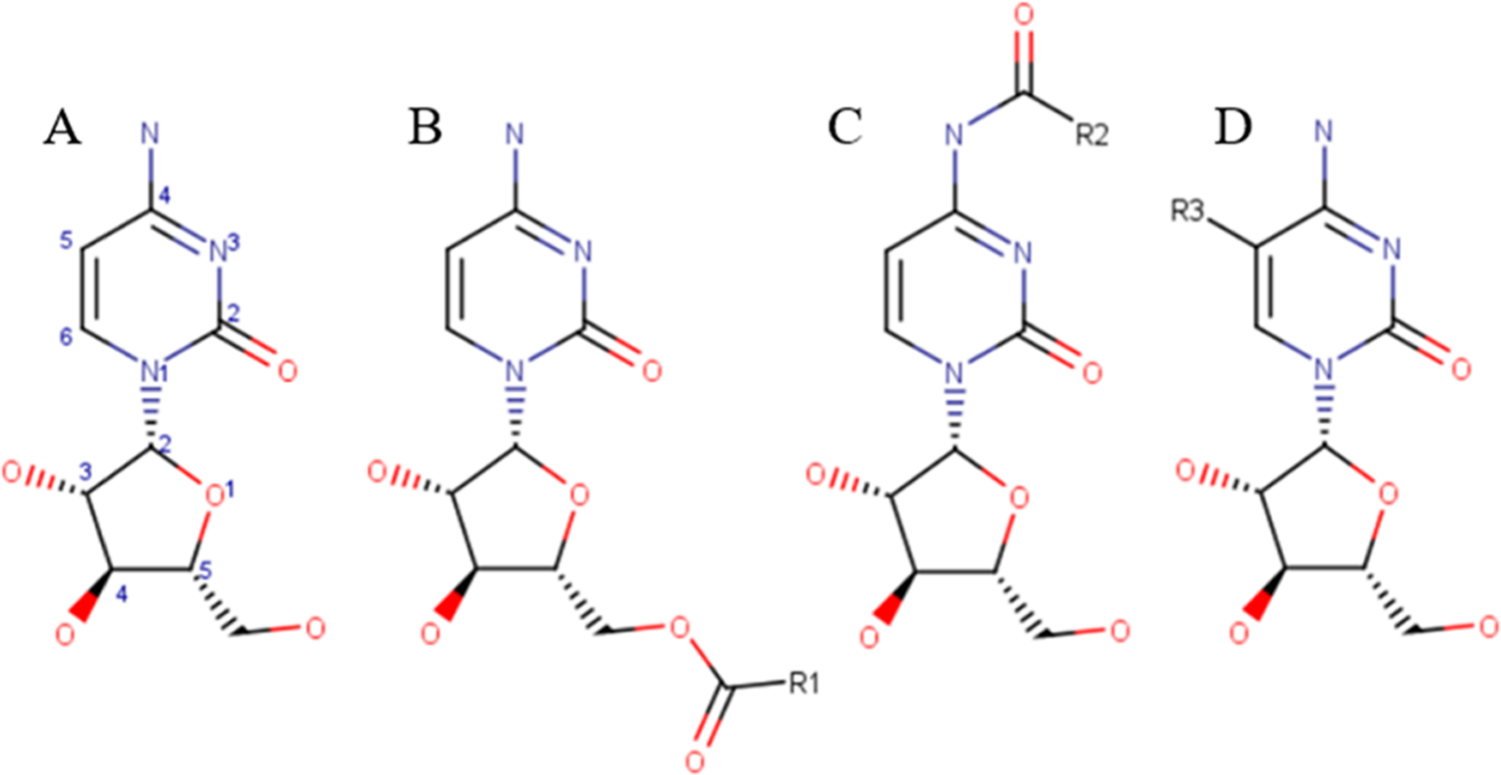
Cytarabine molecule (A) and its ester (B), amide (C), and alkyl (D) prodrugs that were included in a systematic permeation study using COSMOperm calculations. R1 = ethyl (CytO2), butyl (CytO4), hexyl (CytO6), octyl (CytO8), decyl (CytO10), dodecyl (CytO12), tetradecyl (CytO14), and hexadecyl (CytO16); R2 = ethyl (CytN2), butyl (CytN4), hexyl (CytN6), octyl (CytN8), and decyl (CytN10); and R3 = ethyl (CytC2), butyl (CytC4), hexyl (CytC6), octyl (CytC8), and decyl (CytC10).

Each considered molecule was drawn in MarvinSketch 21.9^24^ and saved as a SMILES file. From the SMILES, the LigPrep and MacroModel packages from Schrodinger Release 2017-2 software generated neutral conformers of all compounds in vacuum using the OPLS_2005 force field.^25^ These compounds were cytarabine, its ester, amide, and alkyl chain analogues, as described in Figure 2, and four APIs and their fructose adducts, as shown in Figure 5. For each compound, a maximum of 10 conformers were selected based on MCMM/LMC2 conformation searching algorithm with Monte Carlo structure selection with the MacroModel package from the Schrodinger 2017-2 software suite. The conformers were selected within 5 kcal/mol of the conformer with the lowest energy and RMSD of at least 0.2 nm between individual conformers. A subsequent DFT/B-P/cc-TZVP vacuum and COSMO water optimization with fine grid option were carried out for each conformer using Turbomole 6.3.^26^ The COSMO files for each conformer were obtained from this procedure and subsequently used for partitioning and permeation calculation.

### 2.2 COSMOperm Permeability Calculation

For the purpose of the present work, the membrane with a fixed composition (DPPC/DPPG/cholesterol = 75:10:15 mol %) at 293 K was used as the permeation barrier. The lipid bilayer structure was obtained by molecular dynamics (MD) simulations, as described in detail in our recent work.^6^ Briefly, a membrane bilayer containing 128 preordered lipid molecules was created by an in-house script. Slipids^27^ parameters and the TIP3P^28^ water model were used for the simulation, and the membrane was simulated at 293 K under periodic boundary conditions for approx. 250 ns to ensure the thermodynamic equilibrium of the membrane. The simulation was performed using Gromacs 4.5.4. software.^29^ From the MD simulation, 5 randomly chosen conformations from the last 10 ns were chosen and saved as.pdb files. Using COSMOtherm X8 software and.cosmo files of all membrane components, the five separate.mic files with a σ-profile representation of the membrane sliced into 50 horizontal layers and its electron density and charge of each layer were calculated using the COSMO-RS approach.^30^

**Figure 3:**
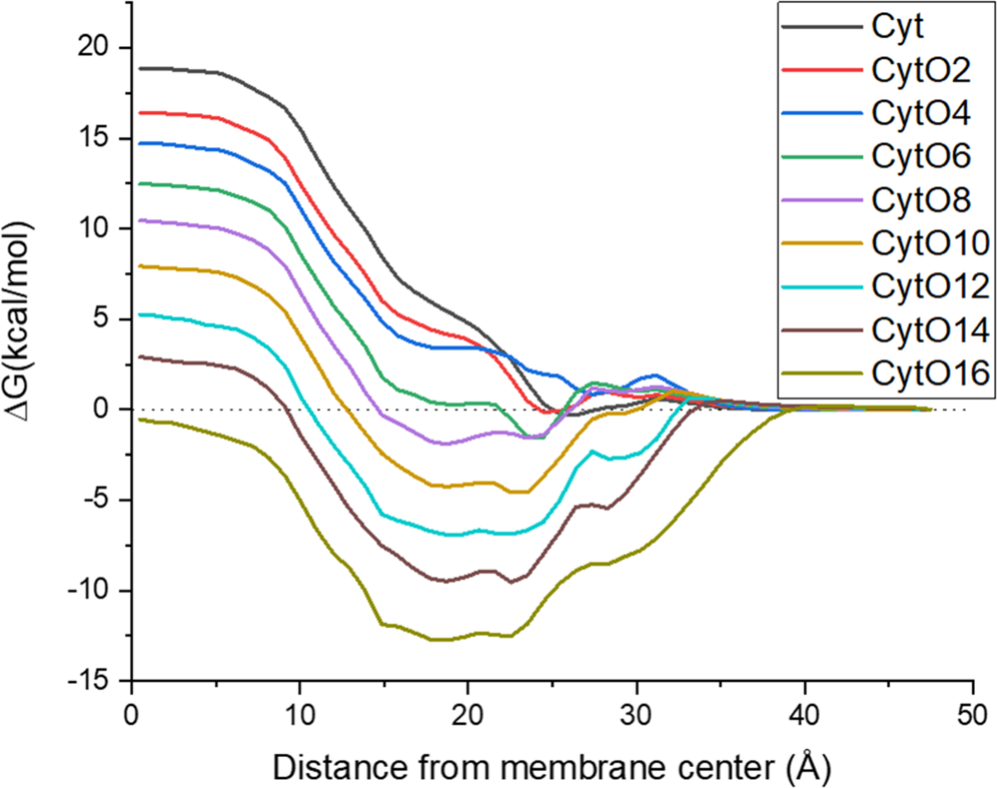
Free energy profiles of cytarabine and its ester prodrugs from acetyl to hexadecyl differing by two carbon lengths between neighboring analogues.

For each combination of the lipid membrane (five snapshots from the DPPC/DPPG/cholesterol membrane) and each conformer of each calculated molecule (26 molecular variants in total), the partition coefficient between the membrane and the water phase was calculated using the COSMOmic^31^ approach, and the permeation rate was calculated using the COSMOperm approach.^32^ Briefly, the chemical potential of the molecule in that part of the membrane was calculated using the σ-profile of a permeating molecule and the σ-profile of a specific layer of the membrane. Therefore, the energy profile through the membrane can be obtained (an example of calculated profiles for several cytarabine prodrugs is shown in Figure 3). Then, the membrane/water partitioning coefficient can be calculated from the energy minima of the chemical potential profile directly using the following equation
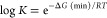
where log* K* is the lipid/water partition coefficient, Δ*G* (min) is the free energy change for permeating molecule between the water environment and the energy minimum, *R* is the universal gas constant, and *T* is the temperature in K. The permeation rate across the liposome membrane was calculated using the Diamond and Katz model for the steady-state flux of a solute through the membrane^33^
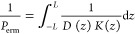
where 2*L* is the thickness of the membrane, *D* (*z*) is the diffusivity of the permeating molecule in the membrane layer, and *K* (*z*) is the partition coefficient of the permeating molecule in the membrane layer. Finally, the results for all investigated molecular structures were incorporated into the MolMeDB database.^34^

## 3 Results and Discussion

### 3.1 Molecule Selection and Case Study Strategy

Recently,^6^ the permeability and partitioning coefficients of 56 APIs from the DrugBank database were calculated and sorted into the LBCS diagram. Out of these, four APIs were identified as particularly poorly permeating: allosamidin, azacitidine, decitabine, and cytarabine. For the sake of case studies in the present work, cytarabine has been selected. Cytarabine is an antineoplastic agent used to treat acute myeloid leukemia, acute lymphocytic leukemia, chronic myelogenous leukemia, meningeal leukemia, and other meningeal neoplasms. Cytarabine is on the WHO list of essential medicines due to its therapeutic significance and is usually administered by i.v. infusion, intrathecal injection, or subcutaneous injection as a solution, but liposomal formulations have also been proposed, e.g., DepoCyte^35^ (now discontinued) or Vyxeos liposomal.^36^ There are numerous cytarabine prodrugs in development. These comprise mostly cytarabine amino acids, cytarabine phosphates, and cytarabine fatty acids,^37^ also considered in this work. Cytarabine amino acid prodrugs improve cytarabine permeability,^38^ and they have already achieved satisfactory results in clinical trials.^39^ Fatty acid conjugation to cytarabine was done with various fatty acid chain lengths, even with some non-common ones like 24-carbon long double acid chain.^40^ When palmitic acid was attached, the octanol partition coefficient increased by 4 orders of magnitude.^41^

As can be seen from the examples above, adding ester-attached fatty acid is one of the common strategies to enhance cytarabine permeability (and bioavailability). However, a rational guideline for the choice of the fatty acid chain length and its position seems to be lacking. Therefore, various lengths of fatty acids were systematically investigated in this study. To ensure the versatility of our approach, the fatty acids were attached to two different cytarabine parts: nitrogen on the benzene ring and oxygen on the fructose C5 carbon. The temperature for the case study was chosen to be 293 K to mimic the temperature of liposomal preparation during the manufacture. To ensure sufficient API retention in liposomes, the API must not leak spontaneously out of the liposome at the temperature at which it is manufactured. There can be applications where also, at the body temperature, the API must not permeate through liposomes instantaneously but slowly over time. Though the absolute values of permeation rate will differ with temperature, the trends and principles for permeation modification drafted in this publication will stay relevant and be modifiable for a specific situation.

### 3.2 Prodrug Design for Permeability Enhancement

The permeability of the unmodified cytarabine molecule obtained by COSMOperm calculation was log *P*_erm_ = −13.2, which is approx. 4–5 orders of magnitude lower than the ideal range for liposomal formulation. Hence, cytarabine has been chosen for the present study as a representative candidate of a poorly permeating substance to test the computational prodrug design methodology. To improve the permeability of such a poorly permeating molecule, prodrugs containing nonpolar aliphatic hydrocarbon chains attached via a hydroxyl or amine group have been systematically created in silico, and their permeability and partitioning coefficients have been calculated.

Cytarabine contains one amine group and three hydroxyl groups (Figure 2A), which can be theoretically used for prodrug preparation. The amine group and the pentose C5′ hydroxyl group (Figures 2B,C) have been chosen due to their opposite locations. To test the robustness of the computational prodrug design approach, the alkylation of the pyrimidine ring at position 5 next to the amine group (Figure 2D) has also been considered. Esters with carboxylic acids containing 2, 4, 6, 8, 10, 12, 14, and 16 carbon atoms and amides with 2, 4, 6, 8, and 10 carbon long carboxylic acid were created computationally as cytarabine prodrugs. Regarding the pyrimidine ring, 2, 4, 6, 8, and 10 carbons were added.

The COSMOperm method is based on calculating the energy profile through the membrane. The energy profile of cytarabine and its ester prodrugs calculated through one of the randomly chosen snapshots from the last 10 ns of the MD simulation of the membrane is shown in Figure 3. With increasing acyl chain length attached to cytarabine, the energy barrier within the membrane monotonically decreases, resulting in higher permeability. Concurrently, the energy minimum located around 20 Å from the membrane center becomes more pronounced with increasing acyl chain length. A lower minimum leads to a higher membrane/water partitioning coefficient. The permeability and partitioning coefficients have been calculated as described in Section 2.2, and the resulting values are summarized in Table 1.

**Table 1:** Calculated Permeability and Partitioning Coefficients for Different Cytarabine Prodrugs through DPPC/DPPG/Chol (75:10:15) Membrane at 293 K, Using the Mean of 5 Calculations through Randomly Chosen MD Snapshots from the Last 10 ns of Simulation

molecule	log *K* (mol/mol)	log *P*_erm_ (cm/s)		molecule	log *K* (mol/mol)	log *P*_erm_ (cm/s)
cytarabine (Cyt)	–0.68 ± 0.17	–13.19 ± 0.04				
substitution on −OH group using esterification				substitution on −NH_2_ group to make amides		
CytO2	–1.08 ± 0.16	–11.43 ± 0.04		CytN2	–1.15 ± 0.26	–11.33 ± 0.03
CytO4	–1.31 ± 0.05	–10.21 ± 0.06		CytN4	–1.35 ± 0.04	–10.18 ± 0.04
CytO6	–0.25 ± 0.25	–8.67 ± 0.10		CytN6	–1.37 ± 0.04	–8.85 ± 0.04
CytO8	0.16 ± 0.57	–7.22 ± 0.17		CytN8	0.00 ± 0.29	–7.42 ± 0.05
CytO10	2.08 ± 0.60	–5.47 ± 0.23		CytN10	1.37 ± 0.36	–5.77 ± 0.06
CytO12	3.90 ± 0.77	–3.55 ± 0.45		alkyl chain substitution on pyrimidine ring		
CytO14	5.64 ± 0.98	–1.93 ± 0.53		CytC2	–0,92 ± 0.11	–11.65 ± 0.05
CytO16	8.05 ± 0.99	0.08 ± 0.28		CytC4	–0.27 ± 0.13	–10.42 ± 0.08
				CytC6	0.47 ± 0.17	–8.78 ± 0.14
				CytC8	1.91 ± 0.22	–7.75 ± 0.21
				CytC10	3.58 ± 0.40	–5.48 ± 0.42

**Figure 4:**
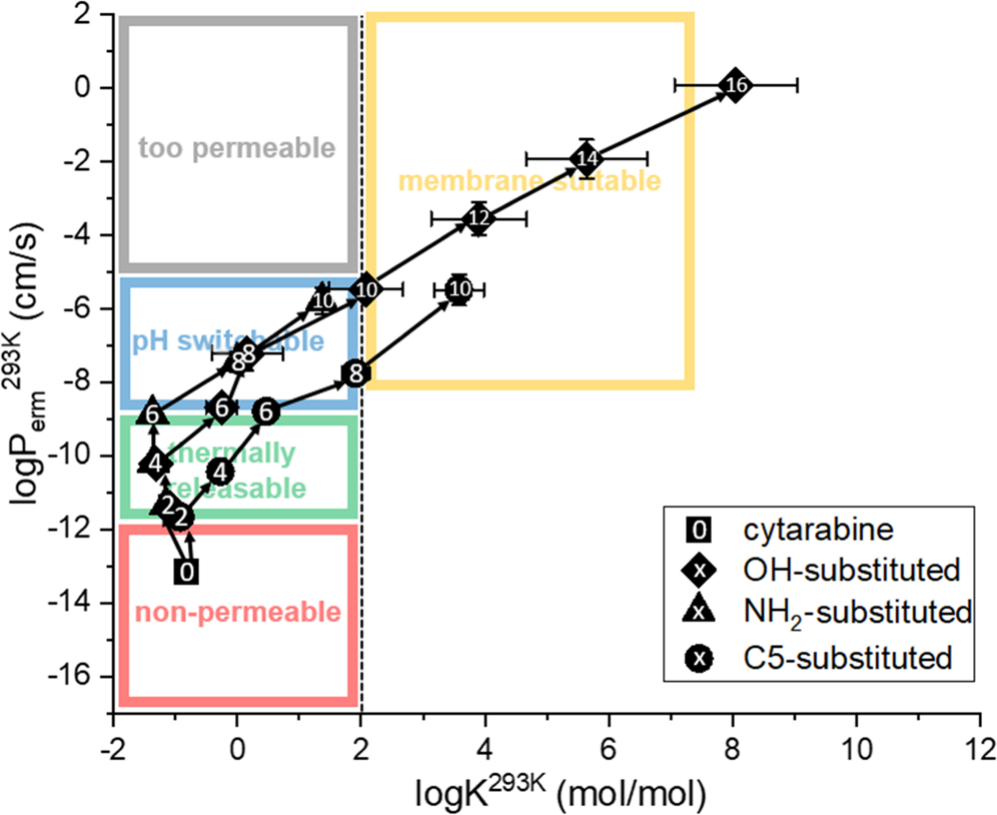
Cytarabine and its prodrugs in LBCS parametric space. The squares, circles, and triangles represent different locations of cytarabine substitution as indicated; the numbers represent the length of the hydrocarbon chain (acyls for O- and N-positions, alkyls for C-position).

The three prodrug families derived from cytarabine show distinct trajectories in the LBCS parametric space of partitioning and permeability coefficient values (Figure 4). It is clearly visible from this graphical representation that even a substance that was initially far from the liposome formulability window can be brought into the feasible range by appropriate modification of its molecular structure. In the specific case of cytarabine, its butyrate prodrugs seem to be the most promising candidates for encapsulation into liposomes and their thermal release. On the other hand, if the formulation needs to be a liposome or lipid particle where the API is supposed to remain dissolved in the membrane, prodrugs with longer hydrocarbon chains (dodecylate, tetradecylate, hexadecylate, or higher) would be the appropriate choice in this case.

It is interesting to observe that while the permeability of the N- and O- and C-substituted prodrugs seems to depend only on the length of the added hydrocarbon chain, the water/membrane partitioning coefficient is also affected by the position of the substitution. From the calculation results, the acetylation of the hydroxyl and the amine group has led to an almost identical increase in the permeation coefficient (1.71 and 1.81 in log *P*_erm_, respectively), and the addition of each further two carbons then resulted in an almost uniform increase in log *P*_erm_ (1.44 ± 0.21 for acyls and 1.52 ± 0.48 for alkyls). The monotonous and nearly linear increase of log *P*_erm_ with the number of carbons added to the original molecular structure continued until the end of the investigated range. A more complex pattern could be seen in the partitioning coefficient. For the first 2–6 carbons added to the structure, there was only a negligible effect on the values of log *K*, followed by the onset of a sustained increase (approx. 1.9 in log *K* for every two carbons).

This observation provides guidelines for the modification of API behavior by the formation of a prodrug: it is possible to increase permeability by several orders of magnitude, either with or without a simultaneous increase in the partitioning coefficient, simply by choosing an appropriate length of the carbon chain and its substitution position.

### 3.3 Prodrug Design for Permeability Reduction

The case discussed above represented a situation where the API permeability was initially too low, and the objective of prodrug design was to improve the permeation. However, if the API permeation rate is too high, such API cannot be successfully encapsulated in liposomes due to premature leakage during manufacturing and storage before administration. The permeation rate through the membrane should be slowed down to prevent excessive spontaneous leakage from liposomes. This can be, in principle, achieved by adding polar groups to the API structure. The choice of possible polar groups is broad, the main limitation being that they should be biocompatible and either not interfere with the pharmacophore or be metabolizable. For the sake of the present work, fructose has been chosen as an example of such a structure.

**Figure 5:**
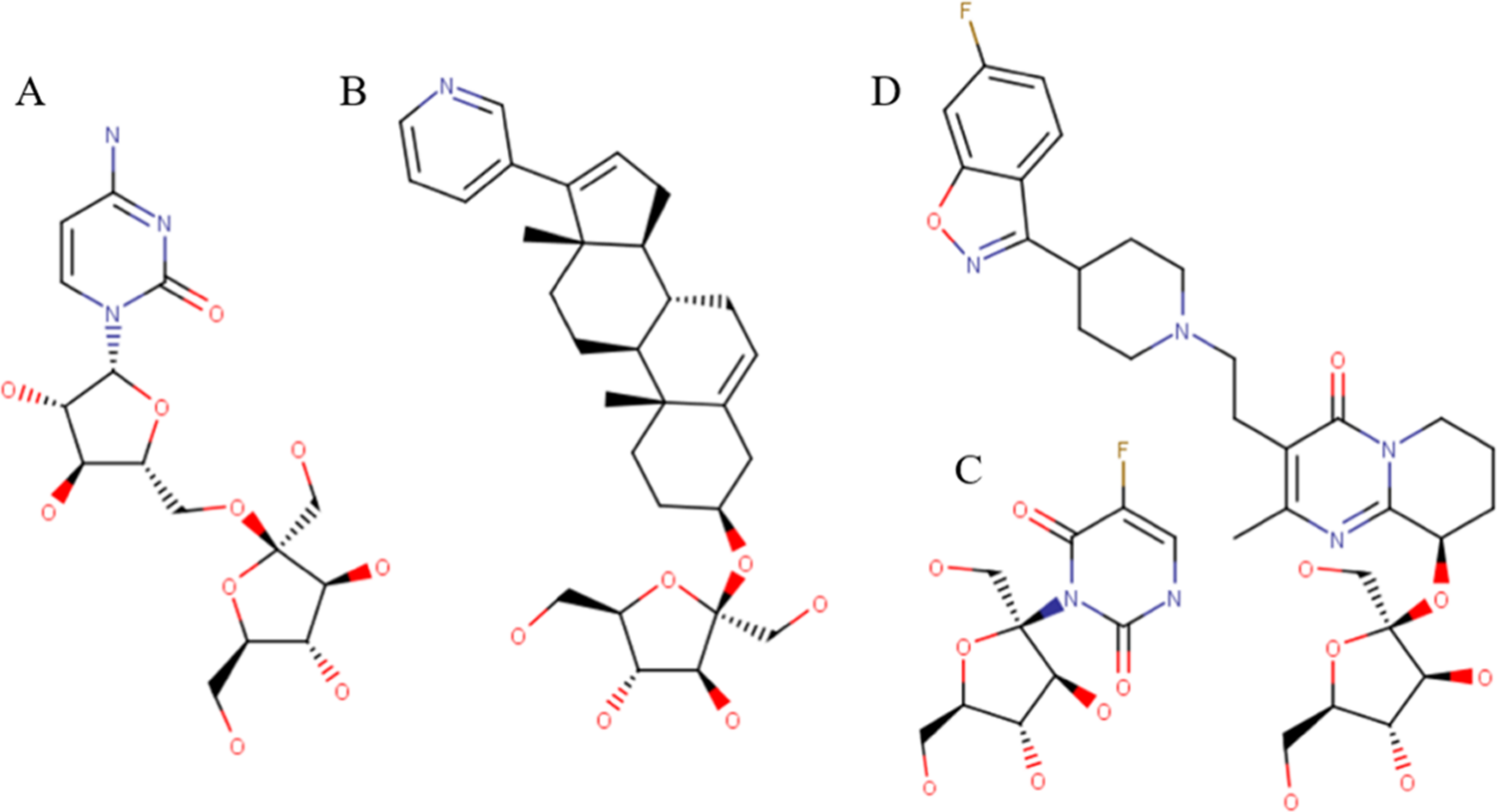
Structures of fructose adducts with cytarabine (A), abiraterone (B), 5-fluorouracil (C), and paliperidone (D).

Four APIs with a different initial location in the LBCS space (i.e., different initial permeability/partitioning combinations) have been selected to demonstrate the computational prodrug design for permeability reduction: anticancer drugs abiraterone, cytarabine, and 5-fluorouracil and an antipsychotic drug paliperidone. Their fructose adducts were formed computationally, and the effect on permeability has been investigated, as described in Section 2.2. The structures are shown in Figure 5.

**Figure 6:**
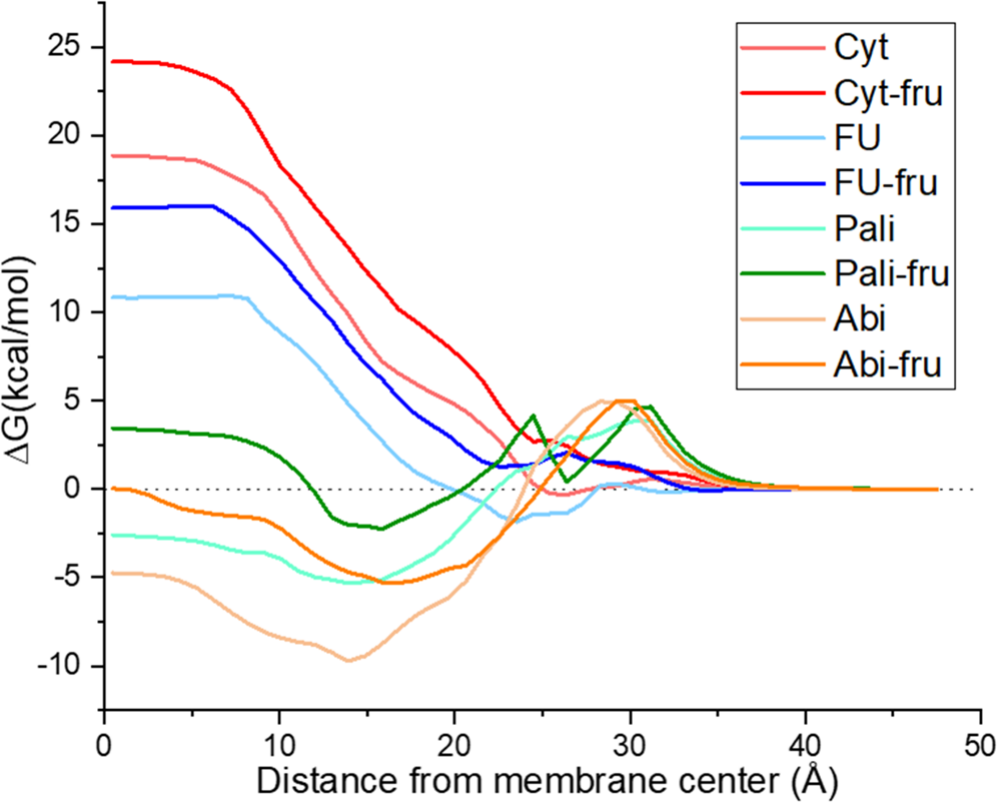
Calculated free energy profiles of the selected molecules and their fructose prodrugs within a lipid bilayer (Abi = abiraterone, FU = 5-fluorouracil, Pali = paliperidone, Cyt = cytarabine, X-fru = fructose prodrug of compound X).

The free energy profiles of native drugs and their fructose adducts, calculated by COSMOperm,^32^ are shown in Figure 6. All fructose adducts generally have an energy barrier higher by approximately 5 kcal/mol than the corresponding free drugs. Although the absolute increase of the energy barrier in the middle of the membrane (position 0 Å in Figure 6) due to fructose addition was identical for all four investigated APIs, the consequence on permeability was not the same. For the two pyrimidine derivates (cytarabine and 5-fluorouracil) that already had a high energy barrier before fructose addition (19 and 11 kcal/mol, respectively) and no or small energy minimum within the membrane, the fructose addition increased the overall energy barrier. Thus, the permeability of both molecules has decreased significantly (by approx. 4 orders of magnitude), as can be seen in Table 2.

**Table 2:** Calculated Partition and Permeation Coefficients for Different APIs and Their Fructose Prodrugs through a DPPC/DPPG/Chol (75:10:15) Membrane at 293 K, Using 5 Calculations through Randomly Chosen MD Snapshots from the Last 10 ns of Simulation

molecule	log *K* (mol/mol)	log *P*_erm_ (cm/s)
cytarabine (Cyt)	–0.68 ± 0.17	–13.19 ± 0.04
Cyt-fru	–1.35 ± 0.05	–17.31 ± 0.04
fluorouracil (FU)	0.69 ± 0.11	–7.28 ± 0.04
FU-fru	–1.13 ± 0.06	–11.21 ± 0.04
abiraterone (Abi)	5.95 ± 0.98	–4.59 ± 0.79
Abi-fru	2.70 ± 0.72	–4.00 ± 0.61
paliperidone (Pali)	2.95 ± 0.43	–4.24 ± 0.58
Pali-fru	0.63 ± 0.38	–6.59 ± 0.97

On the other hand, when considering the two already rapidly permeating molecules (abiraterone and paliperidone), a different pattern was found. Their energy in the membrane center increased by approximately 5 kcal/mol, as in the case of cytarabine and 5-fluorouracil. However, the fructose addition also affected the position and magnitude of an energy minimum within the membrane (at approx. 15–20 Å from the membrane center, as shown in Figure 6). In the case of paliperidone, the minimum was higher by 3 kcal/mol. Therefore, there was still an additional energy barrier which decreased permeability by about 2 orders of magnitude. However, abiraterone showed an increase of the energy minimum by the same amount as in the middle of the membrane, and consequently, the predicted permeation rate of abiraterone and its fructose adduct did not differ significantly.

**Figure 7:**
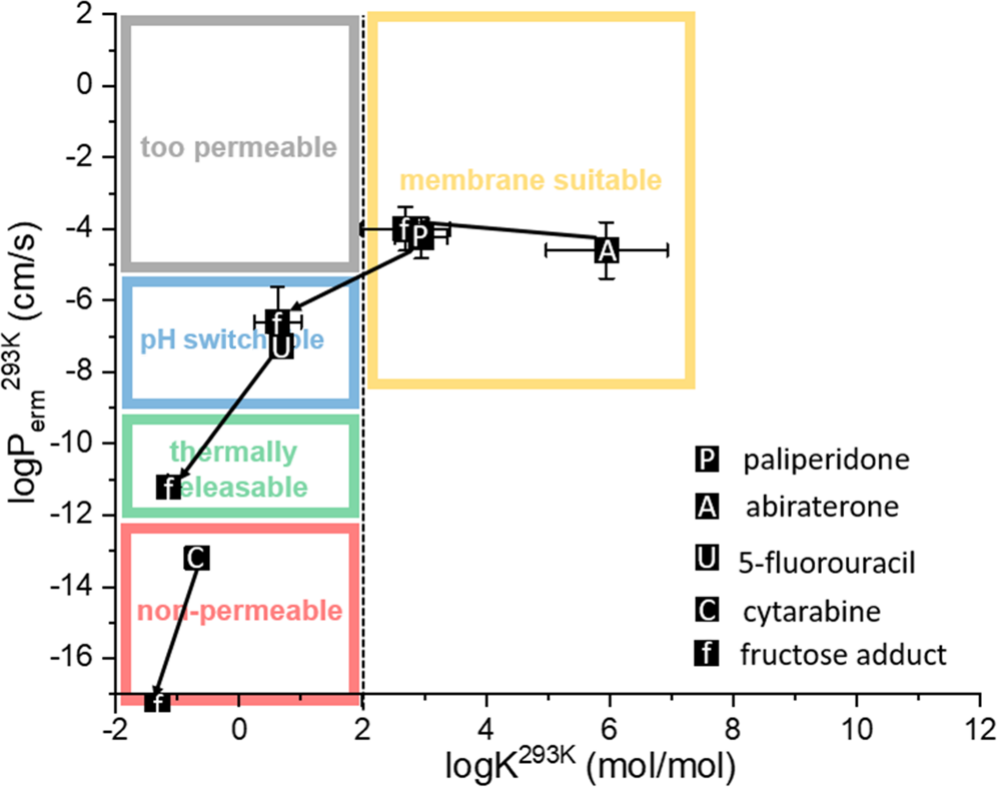
Selected drugs and their corresponding fructose prodrugs in LBCS parametric space as defined in Figure 1. Black squares with ″P/A/U/C″ represent the position of the studied drug in LBCS. Each molecule representing a square leads an arrow to the square, with ″f″ inside representing the corresponding fructose adduct.

When plotting the positions of the original drugs and their fructose prodrugs in the LBCS parametric space (Figure 7), the combined effect of fructose addition on permeability and partitioning coefficient can be visualized. The expected permeability reduction due to the addition of a polar substance (fructose) resulted in a shift along the log *P*_erm_ axis, which was rather uneven depending on the starting structure. As discussed above, cytarabine and 5-fluorouracil show the most significant permeability reduction after fructose addition (4 orders of magnitude), the permeability reduction of paliperidone was intermediate (about 2 orders of magnitude), and the permeability of abiraterone was practically unaffected by fructose addition. Thus, fructose addition would appear to be sufficient for moving 5-fluorouracil into a feasible region in the LBCS space, whereas, for abiraterone and paliperidone, this might not be the case. Regarding the partitioning coefficient, a systematic shift to lower log *K* values can be seen for all four substances, but the extent to which this happened was again uneven. In general, the more hydrophobic the original substance, the more pronounced the effect of fructose addition due to the increased polarity of the adduct. Thus, for abiraterone and paliperidone, the addition of fructose resulted in a reduction of log *K* by 3.2 and 2.3, respectively, whereas for 5-fluorouracil and cytarabine, the log *K* reduction was a more moderate 1.8 and 1.3, respectively (Figure 7 and Table 2).

In summary, these results illustrate that the attachment of fructose to the drug molecule can be an effective strategy to change its liposomal formulation suitability. Depending on the position of the initial molecule in the LBCS space, fructose addition can change either permeability or partitioning coefficient alone or both parameters simultaneously, improving the liposome formulability of the prodrug.

## 4 Conclusions

The ability of small-molecule APIs to be successfully formulated into liposomal carriers intended for parenteral administration depends on the combination of their water/lipid equilibrium partitioning coefficient and permeability across the lipid bilayer. As only a few APIs in their native form fall into the ideal formulability window, the possibilities for permeability and partitioning coefficient modification by a systematic change of the API molecular structure have been investigated in this work. We have investigated the feasibility of moving the position of an API in the LBCS formulation diagram in either direction, i.e., permeability enhancement or permeability suppression using a computational approach based on COSMOperm. We have created a systematic line of prodrugs derived from a poorly permeating API (cytarabine) to study the effects on permeability enhancement by calculating partitioning and permeation coefficients. These prodrugs were esters and amides of cytarabine with various carbon chain lengths. Various carbon chains were also added to compare these results with simple carbon chain addition. A linear dependence of permeability coefficients on the length of a hydrocarbon chain added to the molecule was found, independently of the substitution location. However, the dependence of the membrane partitioning coefficient on the substitution location and the hydrocarbon chain length was not monotonic, especially for shorter carboxyls. Based on the computational results, it was possible to identify potential cytarabine prodrugs that should exhibit enhanced permeability and yet avoid overarching membrane partitioning that would block the drug′s availability to the aqueous phase. This means that using our approach, on-demand permeability can be reached by the addition of a proper length of carboxylic acid to form a prodrug.

Prodrug design strategies for permeability reduction have been investigated as well. From the various possibilities of polar molecules that can be added to the API to form a prodrug, fructose was chosen as an example of a very polar and biocompatible molecule. The effect of adding fructose as a representative polar structure to four APIs with the different initial positions in the LBCS diagram (cytarabine, abiraterone, 5-fluorouracil, and paliperidone) was explored. It was found that although fructose addition resulted in a nearly identical increase of the free energy barrier represented by the lipid bilayer, the effect on permeation rate was strongly API-specific. Three scenarios were identified depending on the initial API: (i) reduction of permeability only, (ii) reduction of partitioning coefficient only, and (iii) simultaneous reduction of both permeability and partitioning coefficient. Therefore, the addition of fructose to API into prodrug formation does not universally lead to permeation reduction. Rather, it is dependent on the API structure and the initial position of the substance in the LBCS diagram.

In summary, a computational methodology for virtual prodrug design has been developed. It has been demonstrated that by systematically modifying the molecular structure of the original API, it is possible to change permeability and partitioning coefficient either simultaneously or individually and thus move the position of the API in the LBCS diagram in the desired direction. Hence, an API with initially unfavorable properties (either too high or too low permeability) can be converted into a prodrug more suitable for a liposomal formulation. Of course, the computational selection of potential prodrug candidates is only the first step, which must be followed by the actual synthesis of the proposed structures, their physicochemical characterization, and pharmacological evaluation.
